# Health-promoting lifestyle and its association with the academic achievements of medical students in Saudi Arabia

**DOI:** 10.12669/pjms.37.2.3417

**Published:** 2021

**Authors:** Mohammed Mahmoud Al-Momani

**Affiliations:** 1 Mohammed Mahmoud Al-Momani, PhD, RN. Department of Community Health Sciences, College of Applied Medical Sciences, King Saud University, Kingdom of Saudi Arabia

**Keywords:** BMI, Lifestyle, Academic achievement, HPLP II score

## Abstract

**Objective:**

This study aimed to assess health-promoting lifestyles among university medical students and to investigate whether such lifestyles are associated with students’ academic achievement.

**Methods:**

A cross-sectional descriptive investigative study was performed on 576 medical college students of King Saud University (KSU) in March 2019. Data were collected using the Health-Promoting Lifestyle Profile II (HPLP II) scale, which includes six dimensions (health responsibility, spiritual growth, physical activity, interpersonal relationships, nutrition, and stress management). The data were analyzed using descriptive and inferential statistics at a significance level of P < 0.05.

**Results:**

A mean score of 3.39 for total health-promoting behaviors was reported. The highest mean score was reported for spiritual growth (3.75) and the lowest was reported for health responsibility (3.23). The mean body mass index (BMI) of the students was 24.9 ± 6.4 kg/m^2^. An analysis of variance identified an association between a student’s GPA and the spiritual growth dimension (P = 0.014). Based on the Pearson matrix correlation coefficient, there was a statistically significant and positive relationship between the HPLP II dimensions (P < 0.05).

**Conclusion:**

Since the HPLP scores were good, there is a need to enhance and promote healthy behaviors in students. The BMI data indicated that one-third of male students were overweight or obese; thus, health program planning, particularly involving physical activity and nutrition, is recommended.

## INTRODUCTION

Approximately 70% of non-communicable diseases, which are commonly known as civilization diseases and include cancers, heart diseases, cerebrovascular accident (CVA), hypertension, diabetes, chronic obstructive pulmonary disease (COPD), and obesity, are associated with an individual’s lifestyle behaviors.^1^ The burden of these diseases is progressively increasing and is considered as the universal biggest killers. Non-communicable diseases continue to be a leading cause of premature death and cause severe disabilities among survivors.^2^ These diseases are challenging for people in developing countries. They can drive them into amplified poverty as a result of their lack of productivity and the need to pay for medications and health care for a prolonged period of time.^3^

Health-promoting lifestyle behaviors can be grouped into six dimensions: regular exercise, a healthy balanced diet, health responsibility, stress management, spiritual growth, and interpersonal relationships.^4^ Implementing positive health-promoting behaviors decreases morbidity and mortality and increases or sustains an individual’s happiness and contentment.^5^

Students’ time at university is a critical period characterized by biological growth, major social role transitions, and health-related behavior changes. Worldwide, studies have indicated that youth are prone to unhealthy lifestyles such as tobacco use, unhealthy diets, increased stress, physical immobility, risky sexual behaviors, injury and violence, etc.^6^ A study at Southern Alabama investigated the health promotion and risk behaviors of college freshmen and found that 25% of the participants had driven after drinking alcohol, 32% had an unhealthy diet and were overweight, 12% were smokers, 34% were sexually active, and 27% used marijuana.^7^ Furthermore, university life is full of impulsivity coexisting with vulnerability, which is affected by peer gatherings and media influence that causes changes in the students’ perceptions and practices with the acquisition of emotional and other skills.^8^

According to the World Health Organization, 60% of a person’s health status and quality of life is dependent on individual’s behavior and lifestyle.^9^ Healthy lifestyles are crucial and are one of the most sensitive indicators influencing students’ academic performance and ensuring they graduate on time. Universities play an important role in producing a conductive environment for healthy lifestyles and in helping students manage their life.^10^

Hence investigating the healthy patterns of lifestyles among medical students is vital because these students would be engaged in health care in various health settings; therefore, they can detect the early signs of risk factors for chronic diseases and create health promotion choices in their patient care accordingly.

## METHODS

This descriptive cross-sectional study was performed in March 2019 among medical students belonging to medicine, pharmacology, nursing, and applied medical sciences faculties at King Saud University (KSU), Riyadh, Saudi Arabia. All undergraduate medical faculties students from the first to sixth academic years were considered eligible. Students who were in an exam period or had any disease were excluded to remove the confounding effects of these factors. Using a convenience sample, the calculated sample size was 576 students. After obtaining ethical approval from the College of Applied Medical Sciences’ research and ethics committee (CAMS 66-36/37), the purpose and objectives of the study were explained to the participants, and their participation was assured to be voluntary. The participants were confirmed for the confidentiality and anonymity of the data.

A two-part self-administered questionnaire was employed. The first part consisted of sociodemographic questions (gender, age, academic year, grade point average (GPA), residence, family size, and monthly income). Body mass index (BMI) was also evaluated, and based on their BMI, the students were categorized as underweight (<18.5 kg/m^2^), normal (18.5–24.9 kg/m^2^), or overweight (>25 kg/m^2^). The second part consisted of the Health-Promoting Lifestyle Profile II (HPLP II) questionnaire. The HPLP II tool measured 52 health-promoting actions that were split into six aspects: health responsibility (nine items), spiritual growth (nine items), physical activity (eight items), interpersonal relationships (nine items), nutrition (nine items), and stress management (eight items). With the aid of a Likert scale, behaviors were scored as follows: never (1), sometimes (2), frequently (3), and routinely (4). The total HPLP II score was further classified into four levels: poor (score of 1–1.73), moderate (score of 1.74–2.48), good (score of 2.49–3.23), and excellent (score of 3.24–4). High scores in every subscale indicated more frequent health-promoting behaviors. The overall scale of the original version of the HPLP II reported a Cronbach’s alpha of 0.87, and the Cronbach’s alpha for the six subscales ranged from 0.76 to 0.84.

In this study, the HPLP II scale was converted into a simplified Arabic version, and two linguists verified the accuracy of the translation. A pilot study was conducted to assess the participants’ ability to complete the translated version. Twenty students completed the Arabic version of the questionnaire to test the clarity and relevance of the statements. All items were understood properly and completed without any difficulties. The questionnaires were distributed among eligible students during academic classes in March 2019 by a trained research assistant.

### Statistical Analysis

The Statistical Package for Social Sciences (SPSS) version 17.0 (IBM Corporation, USA) for windows was used for the data analysis. A descriptive analysis was used to describe the frequencies, means, and standard deviations (SDs) of the participants’ characteristics and HPLP II scores. The scores for the overall health-promoting behavior scale were normally distributed, and its association with academic performance scale was analyzed using a one-way analysis of variance (ANOVA). Moreover, associations between the scores of the healthy lifestyle dimensions were assessed with the Pearson correlation coefficient. The level of significance was set at <0.05.

## RESULTS

Of the 576 completed questionnaires, 312 (54.2%) were completed by males and 64 (45.8%) were completed by females, with an age range of 18–25 years and a mean age of 21.9 ± 1.0 years. Almost 48% participants were in their first and second academic years, followed by 38.7% in their third and fourth academic years. Most of the students (53.3%) had a GPA between 3 and 3.99, and 67.9% of students reported that they lived with their parents. Approximately two-thirds (66.1%) stated that their family size was ≥7 persons. Additionally, 42.9% reported a family income of 10,000–20,000 Saudi Riyal. Table-I describes the detailed results.

**Table-I:** Respondents’ demographic information (n = 576).

*Socio-demographic factors*	*Frequency*	*Percentage*
***Gender***		
Male	312	54.2
Female	264	45.8
***Age (years)***		
18-20	218	37.8
21-23	233	40.5
>23	125	21.7
***Academic year***		
First and Second	275	47.8
Third and Fourth	223	38.7
Fifth or higher	78	13.5
***GPA/out of 5***		
<3	128	22.2
3-3.99	307	53.3
4-4.74	112	19.5
≥4.75	29	5.0
***Residence***		
n campus	67	11.6
Off campus	118	20.5
With parents	391	67.9
***Family size***		
<7	195	33.9
≥7	381	66.1
***Family monthly income/RS***		
<10000	53	9.2
10000-20000	247	42.9
21000-30000	185	32.1
>30000	91	15.8

The mean BMI of the respondents was 24.9 ± 6.4 kg/m2 (range, 14.37 to 36.43 kg/m2). Overall, 51.3% of male students had a BMI in the normal range, 17.6% were underweight, and 31.1% were overweight and/or obese. For females, 63.6% had a BMI in the normal range, 29.1% were underweight, and 7.3% were overweight and/or obese. The mean score for total health-promoting behaviors was 3.39, which was in the good range. The highest mean score was reported in the spiritual growth dimension (3.75), and the lowest score was reported for health responsibility (3.23) (Table-II). An independent sample t-test was conducted to compare the mean scores of the health lifestyle dimensions by gender. The results showed that boys had a higher mean physical activity score than females (P < 0.001). Conversely, females had a higher mean health responsibility score than males (P = 0.082).

**Table-II:** Scores of lifestyle dimensions among participating students by gender.

*Lifestyle dimensions*	*Male*	*Female*	*P-value*	*All subjects*
	
	*Mean (SD)*	*Mean (SD)*		*Mean (SD)*
Health responsibility	3.18 (0.71)	3.28 (0.62)	0.082	3.23 (0.665)
Physical activity	3.12 (0.76)	2.88 (0.68)	<0.001	3.00 (0.681)
Nutrition behavior	3.16 (0.56)	3.32 (0.63)	0.681	3.24 (0.595)
Spiritual growth	3.82 (0.72)	3.67 (0.66)	0.219	3.75 (0.69)
Interpersonal relationships	3.51 (0.64)	3.46 (0.58)	0.392	3.48 (0.61)
Stress management	3.62 (0.68)	3.56 (0.62)	0.604	3.59 (0.65)
Overall lifestyle	3.33 (0.61)	3.37 (0.58)	0.416	3.39 (0.49)

The associations between lifestyle dimensions and GPA are displayed in Table-III. The analysis of variance revealed no associations between students’ GPA and lifestyle dimensions except for spiritual growth (P = 0.014). A higher GPA tended to be associated with higher spiritual growth.

**Table-III:** Scores of lifestyle dimensions among participating students by GPA.

*Lifestyle dimensions*	*Students’ GPA*				

	*<3.00*	*3-3.99*	*4-4.75*	*≥4.75*	*P-value*

	*Mean (SD)*	*Mean (SD)*	*Mean (SD)*	*Mean (SD)*	
Health responsibility	3.20 (0.61	3.22 (0.67)	3.24 (0.58)	3.25 (0.72)	0.438
Physical activity	3.02 (0.95)	2.97 (0.72)	3.01 (0.66)	3.04 (0.61)	0.647
Nutrition behavior	3.22 (0.67)	3.28 (0.61)	3.26 (0.52)	3.21 (0.74)	0.315
Spiritual growth	3.60 (0.66)	3.76 (0.68)	3.80 (0.73)	3.89 (0.81)	0.014*
Interpersonal relationships	3.62 (0.53)	3.38 (0.71)	3.40 (0.49)	3.52 (0.71)	0.761
Stress management	3.51 (0.72)	3.59 (0.89)	3.62 (0.69)	3.67 (0.74)	0.132

Based on the Pearson matrix correlation coefficient, there were statistically significant and positive relationships between HPLP dimensions (P < 0.01). There was a moderate relationship between nutrition behavior and personal health responsibility (r = 0.564) and physical activity (r = 0.488) (Table-IV).

**Table-IV:** Pearson correlation matrix (r coefficient) of health lifestyle dimensions.

*Lifestyle dimensions*	*D1*	*D2*	*D3*	*D4*	*D5*
Health responsibility	1				
Physical activity	0.390**	0.380**	1		
Nutrition behavior	0.630**		0.310**	0.370**	0.249**	0.316**	0.381	1
Spiritual growth	0.283**	0.278**			0.330**	0.296**		
Interpersonal relationships	0.291**		0.488**			
Stress management	0.564**				

** Correlation is significant at the level of 0.01

## DISCUSSION

Inappropriate lifestyles and behaviors play a vital role in the development of many non-communicable diseases. Our study results provided a hint of the lifestyles present among medical students in Saudi Arabia. University students are the future decision-makers for organizations, communities, and countries. Considering the importance of their health, supporting health-promoting behaviors among Saudi Arabian students should be a priority for policymakers and health care professionals by providing community-based services aimed at helping these students develop a healthy lifestyle.

The study findings revealed that the students’ mean health-promoting lifestyle score was 3.39 ± 0.76. This finding suggests that students tend to display good health-promoting behaviors. This is consistent with previous findings.^8^,^11^-^13^ However, a study conducted by Al Zahrani et al.^14^ at Saudi University revealed a moderate total HPLP II score among medical students. These slightly different might be ascribed to the sample type and sample size. Our study’s findings indicated the importance of health promotion planning with an emphasis on empowerment to develop a healthy lifestyle among this target group.

When assessing the health-promoting lifestyle dimensions, the students scored the highest in spiritual growth and stress management but the lowest for physical activity, health responsibility, and nutrition. These results are somewhat similar to the findings of other studies.^6^,^15^,^16^

In this study, spiritual growth received the highest score among all health-promoting lifestyle dimensions. This finding is consistent with studies by Al Khwaldeh^15^ and Can et al.^17^ No differences between male and female students were found in terms of spiritual growth. The impact of the culture and belief system of the Saudi Arabian society and the university environment might contribute to maintaining spiritual growth.

Students in our study reported good scores in stress management. They follow techniques and methods that can help reduce stress such as relaxation and meditation, getting enough sleep and concentrating on pleasant thoughts at bedtime, and maintaining balance between studying and relaxing time.

The results showed weak performances in physical activity and nutrition habits. In the US and Europe, university students are frequently engaged in health-promoting behaviors such as consuming a healthy diet and performing physical activity; hence, most research on health-promoting behaviors has been commenced in these countries.^18^,^19^

Gender differences in the analysis indicated that females engaged in a significantly lower level of exercise compared with males. This result is consistent with those reported by Mehri^5^ and by Burke & McCarthy.^20^ This could be explained by the effects of culture on physical activity. Girls in Saudi Arabia have low self-efficacy in performing outdoor activities and other visible exercise opportunities. The food intake patterns of the university students are of specific concern because they tend to skip meals frequently and consume fast foods and snacks.

Female students were more likely to consume healthy foods than males. This result is consistent with the findings of a study by Wang et al.^9^ This could be explained because almost all females were living with their parents who tended to consume healthy food. We also found that health responsibilities received the lowest score among the six health lifestyle dimensions. The undergraduate students who reside on campus and off-campus with classmates are unlikely to care about their health as much as students living with their parents who frequently remind the students about health behaviors.^9^ Moreover, university life augments stress and demands independent decision making. The results showed that female’s students are likely to watch TV programs about improving their fitness and health, and they are more likely to follow health care advice and discuss their concerns; they also weigh themselves at least monthly to assess any changes.

Although approximately one-third of the men were overweight based on their BMI measurements, less than 10% of the females were overweight. Previous research has revealed that there is an increasing prevalence of overweight and obesity in both developing and developed countries.^21^ Studies conducted on the Saudi Arabian population have revealed that the prevalence of obesity and overweight is high across both genders. Males tend to gain weight than female in Saudi Arabia.^22^,^23^

BMI may be considered a trigger that urges individuals to necessitate some strategies for modifying their lifestyle. Considering the importance of body image and its relationship with self-concept, particularly among females, BMI may become a focal point when designing health education/promotion programs for students. Moreover, there was an increased risk of being overweight or obese among married and off-campus students. Thus, health-promoting interventions should be increasingly focused on these student groups.

There was no association between the students’ GPA and lifestyle dimensions except for spiritual growth. This result is in line with a study conducted by Bakoue et al. who believed that spiritual growth has an impact on the daily life of people, making them less worried and anxious.^24^ Therefore, it enhances their cognitive skills such as concentration and attention and allows them to solve their problems properly.^25^

The Pearson correlation matrix exhibited a positive and noteworthy correlation between the elements of health-promoting behaviors. Notably, there is interrelationship between aspects of behaviors that help in maintaining and promoting health, and health promoting behaviors may have a synergistic effect on other behaviors. This finding is consistent with the study by Bastani.^8^

The results of the present study showed that health stress control had a strong relationship with physical activity and nutrition. In other words, the better the stress management among the students, the better their physical activity and nutrition habits. Thus, developing and executing goal-oriented programs to promote stress management may promote physical activity and good nutritional behaviors among students. Stress control focuses on the methods and techniques used to control and manage stress.

### Limitation of the study

The cross-sectional design of this study does not explain causation and changes that occur over time in lifestyle behaviors among these students. Therefore, the findings should be generalized with caution. Because all the information collected in this study was based on self-reporting, the answers may have a socially desirable response bias.

## CONCLUSIONS

The good HPLP scores among the students highlight the need for enhancing and promoting their healthy behaviors, with a particular focus on physical activity and nutrition behaviors. Since more than one-third of the male students were overweight or obese, program planning to improve their health behaviors is recommended.
